# Elongation factor Tu on *Escherichia coli* isolated from urine of kidney stone patients promotes calcium oxalate crystal growth and aggregation

**DOI:** 10.1038/s41598-017-03213-x

**Published:** 2017-06-07

**Authors:** Piyawan Amimanan, Ratree Tavichakorntrakool, Kedsarin Fong-ngern, Pipat Sribenjalux, Aroonlug Lulitanond, Vitoon Prasongwatana, Chaisiri Wongkham, Patcharee Boonsiri, Jariya Umka Welbat, Visith Thongboonkerd

**Affiliations:** 1 Faculty of Graduate School, Khon Kaen University, Khon Kaen, Thailand; 2 Centre for Research and Development of Medical Diagnostic Laboratories, Faculty of Associated Medical Sciences, Khon Kaen University, Khon Kaen, Thailand; 3 Department of Clinical Microbiology, Faculty of Associated Medical Sciences, Khon Kaen University, Khon Kaen, Thailand; 4 Medical Proteomics Unit, Office for Research and Development, Faculty of Medicine, Siriraj Hospital, Mahidol University, Bangkok, Thailand; 5 Department of Biochemistry, Faculty of Medicine, Khon Kaen University, Khon Kaen, Thailand; 6 Department of Anatomy, Faculty of Medicine, Khon Kaen University, Khon Kaen, Thailand; 7 Center for Research in Complex Systems Science, Mahidol University, Bangkok, Thailand

## Abstract

*Escherichia coli* is the most common bacterium isolated from urine and stone matrix of calcium oxalate (CaOx) stone formers. Whether it has pathogenic role(s) in kidney stone formation or is only entrapped inside the stone remains unclear. We thus evaluated differences between *E. coli* isolated from urine of patients with kidney stone (EUK) and that from patients with urinary tract infection (UTI) without stone (EUU). From 100 stone formers and 200 UTI patients, only four pairs of EUK/EUU isolates had identical antimicrobial susceptibility patterns. Proteomic analysis revealed nine common differentially expressed proteins. Among these, the greater level of elongation factor Tu (EF-Tu) in EUK was validated by Western blotting. Outer membrane vesicles (OMVs) derived from EUK had greater promoting activities on CaOx crystallization, crystal growth and aggregation as compared to those derived from EUU. Neutralizing the OMVs of EUK with monoclonal anti-EF-Tu antibody, not with an isotype antibody, significantly reduced all these OMVs-induced promoting effects. Moreover, immunofluorescence staining of EF-Tu on bacterial cell surface confirmed the greater expression of surface EF-Tu on EUK (vs. EUU). Our data indicate that surface EF-Tu and OMVs play significant roles in promoting activities of *E. coli* on CaOx crystallization, crystal growth and aggregation.

## Introduction

Among all kidney stone types, calcium oxalate (CaOx) stone is the most common one of which the etiology remains unclear^1^. Urinary tract infection (UTI) is known to be associated with kidney stone disease, especially magnesium ammonium phosphate (struvite) type that is the result of infection by urea-splitting bacteria, such as *Proteus mirabilis* ^2–4^. Nevertheless, our recent study has demonstrated that not only struvite but also metabolic stones (i.e. CaOx) are associated with UTI^5^. Moreover, *E. coli*, not *P. mirabilis*, is the most common bacterium found in the urine and stone matrix of the stone formers^5^. However, whether *E. coli* has a pathogenic role in kidney stone formation or is only entrapped inside the stone matrix remains to be elucidated. Interestingly, previous *in vitro* and *in vivo* studies have shown that *E. coli* can promote CaOx crystal growth and aggregation^6, 7^, both of which are the important processes of kidney stone formation. However, the mechanisms underlying such promoting activities of *E. coli* on CaOx crystal growth and aggregation remain unclear.

Recently, proteomics has been widely used to address pathogenic and cellular mechanisms of many various diseases^8, 9^. In the present study, we thus applied a proteomics-based approach to address differences between *E. coli* isolated from urine of patients with kidney stone (EUK) and that isolated from patients with urinary tract infection (UTI) without stone (EUU). Various functional investigations were then performed to address significant roles of the differentially expressed proteins identified from EUK vs. EUU.

## Results & Discussion

From a total of 100 stone formers, nine had positive culture for *E. coli* in their urine (EUK). Antimicrobial susceptibility patterns of these EUK isolates are summarized in Supplementary Table S1. In addition, *E. coli* was also isolated from 200 UTI patients (EUU) who had no kidney stone disease, renal failure, and kidney tumors. Among the latter 200 EUU isolates, only four had identical antimicrobial susceptibility patterns as compared to the other four of the EUK group (Patterns #1–4) (Supplementary Table S2). According to the selection criteria, only these four pairs of EUU and EUK with identical antimicrobial susceptibility patterns (as to reduce the confounding factors that would lead to identification of differentially expressed proteins that were not relevant to our model (e.g., those related to antimicrobial resistance, but not to the stone pathogenesis) were subsequently analyzed for their phenotypic characterizations and differential cellular proteome profiles, followed by functional investigations.

Phenotypic characteristics, including bacterial colony size, cell length and time to mid-log phase of the growth curve, which may be the important factors for adaptive response of bacteria to survive within different environments (e.g., inside vs. outside the stone matrix)^10^, were examined. The findings showed no significant differences of these physical characteristics in EUU vs. EUK groups (Table 1).

**Table 1:** Phenotypic characterizations, including bacterial colony size, cell length and time to mid-log phase of the growth curve of EUU vs. EUK groups.

Parameters	EUU group (Mean ± SEM)	EUK group (Mean ± SEM)	P-value
Bacterial colony size (mm)	2.68 ± 0.04	2.63 ± 0.05	0.45
	(n = 374)	(n = 355)	
Bacterial cell length (μm)	1.91 ± 0.03	1.98 ± 0.02	0.09
	(n = 279)	(n = 291)	
Time to mid-log phase for growth curves (h)	4.33 ± 0.15	4.60 ± 0.28	0.41
	(n = 12)	(n = 12)	

**Figure 1:**
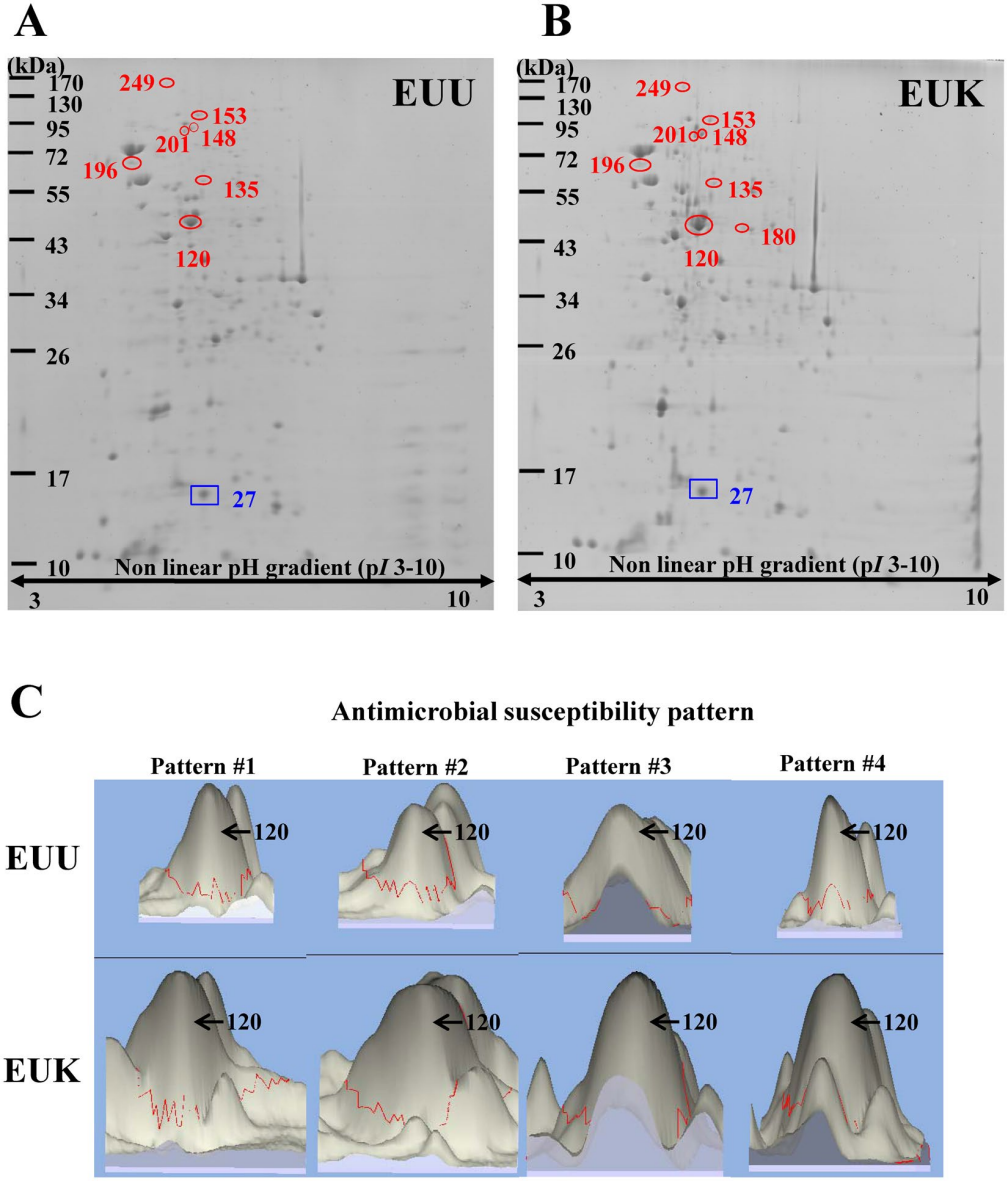
2-D proteome map of the common differentially expressed proteins that were consistent in all four pairs of EUU vs. EUK isolates. (**A**) Representative 2-D gel of EUU isolates. (**B**) Representative 2-D gel of EUK isolates. The circles indicate proteins that were predominant in EUK, whereas a rectangle localizes protein that was predominant in EUU group. These common differences were then subjected to protein identification by nanoLC-MS/MS analysis (see Table 2). (**C**) 3-D zoom-in images demonstrating differential intensity volumes of spot no. 120, which was identified as EF-Tu, in EUU compared to EUK in all four antimicrobial susceptibility patterns.

Cellular proteome of each of the EUU/EUK pairs was then analyzed by 2-DE (n = 3 gels derived from each of EUU and EUK isolates; a total of 24 gels were analyzed in this study). Using colloidal Coomassie blue G-250 staining, approximately 350 protein spots were visualized in each 2-D gel. Spot matching and quantitative intensity analysis together with statistical analysis were performed to define differentially expressed protein spots in each pair of EUU vs. EUK isolates. Among these, the common differences consistent across all the four pairs were our main focus in the present study and were thus subjected to in-gel tryptic digestion and identification by nanoLC-MS/MS (Fig. 1 and Table 2).

**Table 2:** Summary of differentially expressed proteins in EUU vs. EUK that were consistent in all antimicrobial susceptibility patterns.

Spot no.	Protein name	NCBI ID	Identification score (MS/MS ions score)	%Cov	No. of distinct matched peptides	p*I*	MW (kDa)	Spot intensity (arbitrary unit) (mean ± SEM)		Ratio (EUK/EUU)	P-value
								EUU	EUK		
27	Global DNA-binding transcriptional dual regulator H-NS	gi|15801465	1061	66	11	5.43	15.59	1.5950 ± 0.1713	0.8115 ± 0.0574	0.51	0.002
120	Elongation factor Tu	gi|15803852	1666	85	27	5.30	43.43	1.7768 ± 0.1559	3.5646 ± 0.2452	2.00	<0.001
135	Alkyl hydroperoxide reductase subunit F	gi|485774069	2551	65	29	5.47	56.50	0.0912 ± 0.0088	0.2367 ± 0.0203	2.60	<0.001
148	ATP-dependent chaperone protein ClpB	gi|487363647	2772	65	48	5.33	95.63	0.1231 ± 0.0274	0.2605 ± 0.0302	2.12	0.007
153	Pyruvate dehydrogenase E1 component	gi|545300671	2542	58	47	5.46	99.96	0.1662 ± 0.0201	0.4101 ± 0.0523	2.47	0.001
180	Acetate kinase	gi|15832434	1261	67	18	5.85	43.61	0.0000 ± 0.0000	0.1761 ± 0.0202	#DIV/0	<0.001
196	Phosphoenolpyruvate-protein phosphotransferase	gi|15802949	2146	69	37	4.78	63.72	0.3016 ± 0.0112	0.4619 ± 0.1014	1.53	0.048
201	Glycyl-tRNA synthetase beta subunit	gi|487363647	616	63	41	5.26	76.88	0.0866 ± 0.0097	0.1578 ± 0.0218	1.82	0.014
249	DNA-directed RNA polymerase subunit beta	gi|485809349	3463	53	69	5.15	150.95	0.0737 ± 0.0227	0.3758 ± 0.0649	5.10	0.002

These common differentially expressed proteins were identified by nanoLC-MS/MS analysis.

NCBI = National Center for Biotechnology Information

%Cov = %Sequence coverage [(number of the matched residues/total number of residues in the entire sequence) × 100%]

#DIV/0 = Divided by zero.

The common differentially expressed proteins included elongation factor Tu (EF-Tu), alkyl hydroperoxide reductase subunit F (AhpF), ATP-dependent chaperone protein ClpB (clpB), pyruvate dehydrogenase E1 component (AceE), acetate kinase (AckA), phosphoenolpyruvate protein phosphotransferase (ptsI), glycyl-tRNA synthetase beta subunit (glys) and DNA-directed RNA polymerase subunit beta (rpoB) that were predominant in EUK, and global DNA-binding transcriptional dual regulator H-NS (hns) that was predominant in EUU (Table 2). Using UniProt (http://www.uniport.org/help/entry_status), all these identified proteins were classified into four groups, including those involved in carbohydrate metabolism, stress response, protein metabolism, and RNA metabolism (Table 3).

**Table 3:** Functional category and subcellular localization of the common differentially expressed proteins in EUU vs. EUK.

Function/protein name	Spot no.	Subcellular localization	Alteration
***Carbohydrate metabolism***			
Pyruvate dehydrogenase E1 component (AceE)	153	Cytoplasm	Predominate in EUK
Acetate kinase (AckA)	180	Cytoplasm	Detectable only in EUK
Phosphoenolpyruvate-protein phosphotransferase (ptsI)	196	Cytoplasm	Predominate in EUK
***Stress response***			
ATP - dependent chaperone protein ClpB (ClpB)	148	Cytoplasm	Predominate in EUK
***Protein metabolism***			
Elongation factor Tu (EF-Tu)	120	Cytoplasm	Predominate in EUK
Glycyl-tRNA synthetase beta subunit (glys)	201	Cytoplasm	Predominate in EUK
Alkyl hydroperoxide reductase subunit F (AhpF)	135	Cytoplasm	Predominate in EUK
***RNA metabolism***			
DNA-directed RNA polymerase subunit beta (rpoB)	249	Cytoplasm	Predominate in EUK
Global DNA-binding transcriptional dual-regulator H-NS (hns)	27	Cytoplasm	Predominate in EUU

**Figure 2:**
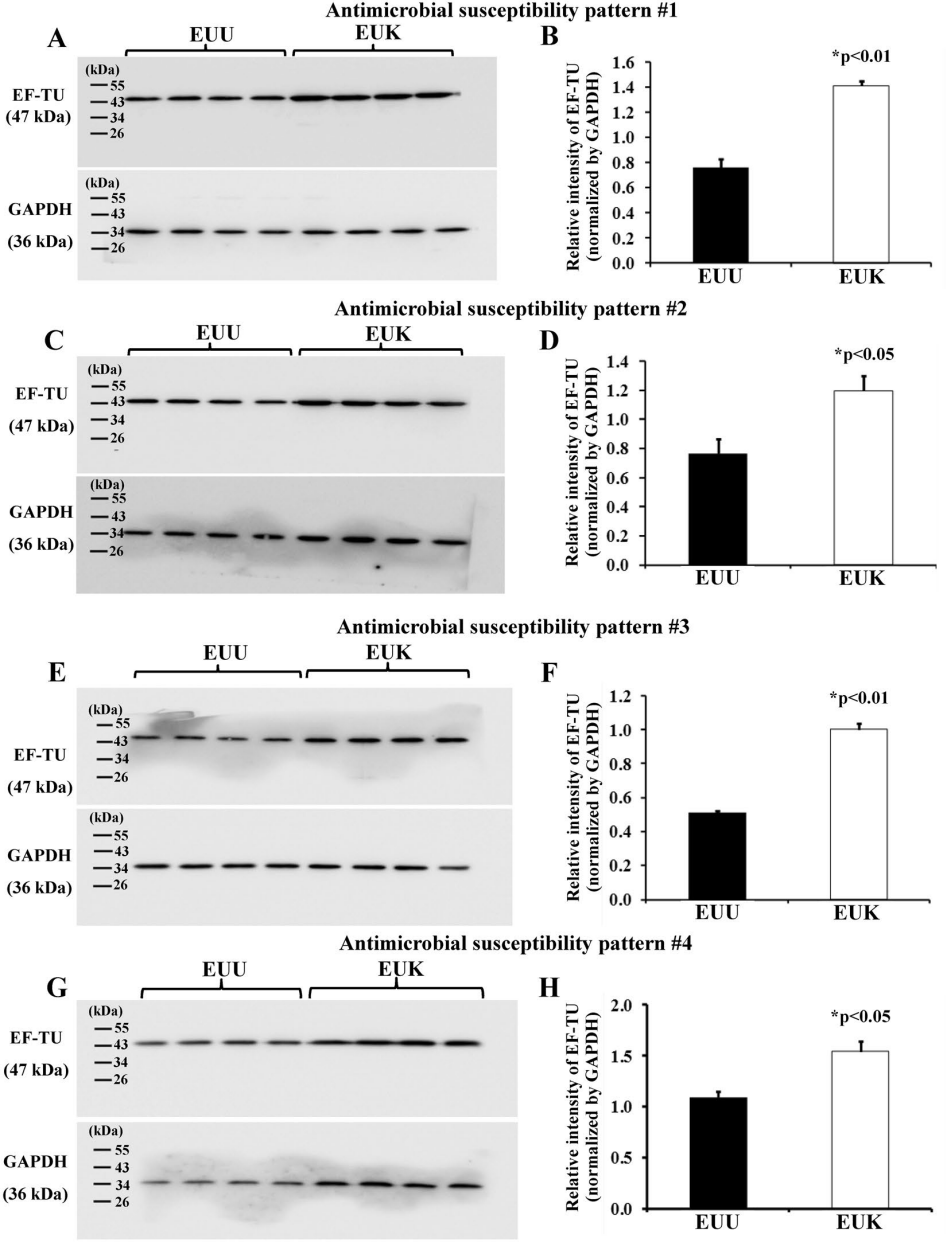
Validation of the proteomic data by Western blot analysis. (**A**,**C**,**E**,**G**) The significantly greater level of EF-Tu in whole cell lysate of EUK as compared to EUU was confirmed in all four pairs of EUU vs. EUK isolates (antimicrobial susceptibility patterns #1–4). GAPDH served as the loading control. (**B**,**D**,**F**,**H**) Relative band intensity level of EF-Tu normalized by GAPDH in EUU vs. EUK. Each bar represented mean ± SEM of the data obtained from four biological replicates in each isolate.

Among the differentially expressed proteins, EF-Tu was the most abundant protein with 2-fold greater level in EUK as compared to EUU (Fig. 1C and Table 2). EF-Tu is a multifunction protein that plays many important roles in (i) transporting codon-specified aminoacyl-tRNA to the A site of the ribosome, (ii) helping chaperones to protect other proteins from aggregation, and (iii) catalyzing disulfide bond formation, isomerization and reduction of proteins^11–13^. In addition, EF-Tu can aggregate and precipitate vinblastine and calcium ions, and then bind to DNase I^14^. Therefore, it may have actin-like properties^15^. Bacterial EF-Tu has been reported to be present in decalcified nanoparticles (NPs) derived from human kidney stones as determined by a proteomics approach^16^. It was thus selected for validation of the proteomic quantitative data by other technique (i.e. Western blotting) and subsequent functional investigations (for its potential role in kidney stone pathogenesis). Western blot analysis confirmed the greater level of EF-Tu in EUK in all four pairs of EUU/EUK with identical antimicrobial susceptibility patterns, whereas GAPDH served as the loading control (Fig. 2). Our data demonstrated the greater level of EF-Tu in EUK as compared to EUU, implicating the potential role of EF-Tu in kidney stone formation and development.

Although EF-Tu has been predicted to be expressed mainly in bacterial cytoplasm (Table 3), it has been also found in native OMVs derived from *E. coli* in another previous proteomics-based study^17^. OMVs that are released from Gram-negative bacteria play important roles in toxin delivery into the host cells and in modulation of immune response in the host^18^. Moreover, another proteomics study has also demonstrated that bacterial EF-Tu is also expressed on OMVs that are immunogenic during *Burkholderia* infection in the murine model^19^. Additionally, Dallo and coworker^20^ have demonstrated that EF-Tu derived from *Acinetobacter baumannii* is associated with the bacterial cell surface, OMVs and fibronectin. Therefore, it is most likely that EF-Tu on the surface and OMVs of pathogenic bacteria are involved in the pathogenesis of bacterial infection by its property to adhere with the host cells and its modulatory effects on the host immune system^21^. Taken together, we thus hypothesize that EF-Tu from *E. coli*, in particular EUK, is also involved in kidney stone pathogenesis via its expression on OMVs that can trigger subsequent phenomena, especially promotion of CaOx crystallization, crystal growth, and crystal aggregation.

**Figure 3:**
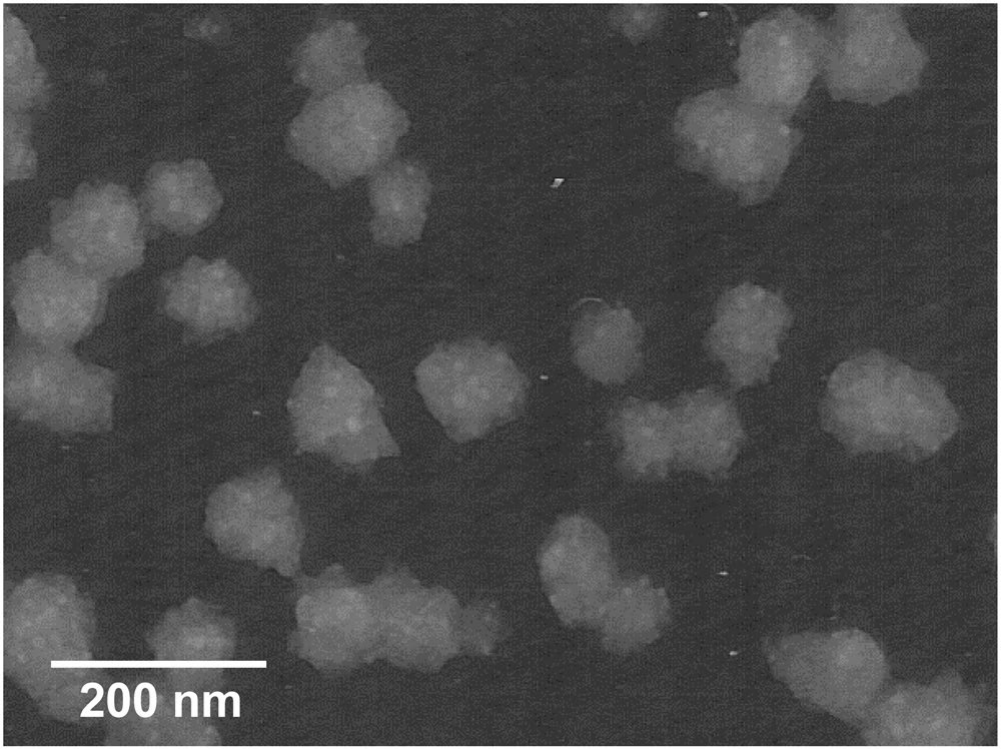
Transmission electron microscopic examination of purified OMVs derived from EUK.

**Figure 4:**
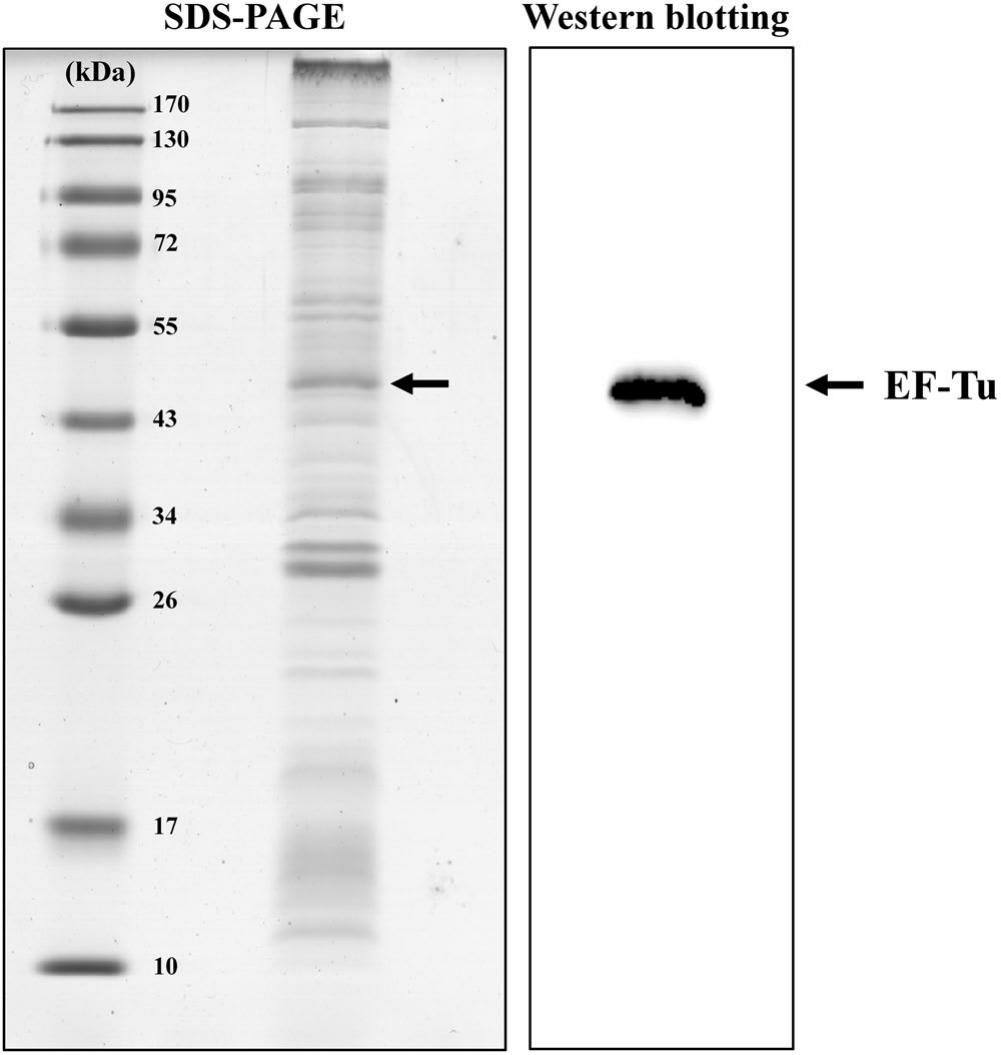
Confirmation of EF-Tu expression on OMVs derived from EUK. Proteins derived from OMVs were resolved by 12% SDS-PAGE and visualized by Coomassie blue G250 staining. Another gel was run in parallel and proteins were transferred onto a PVDF membrane and subjected to Western blot analysis using mouse monoclonal anti-EF-Tu as the primary antibody. The arrow indicates the immunoreactive band of EF-Tu.

To address our hypothesis, OMVs were purified from the culture supernatants derived from EUK isolates. Their typical spherical morphology and appropriate size range (80–90 nm) were confirmed by transmission electron microscope (TEM) (Fig. 3). The expression of EF-Tu on the OMVs of EUK was verified by SDS-PAGE and validated by Western blotting (Fig. 4). The results confirmed that EF-Tu was present on OMVs derived from *E. coli*.

**Figure 5:**
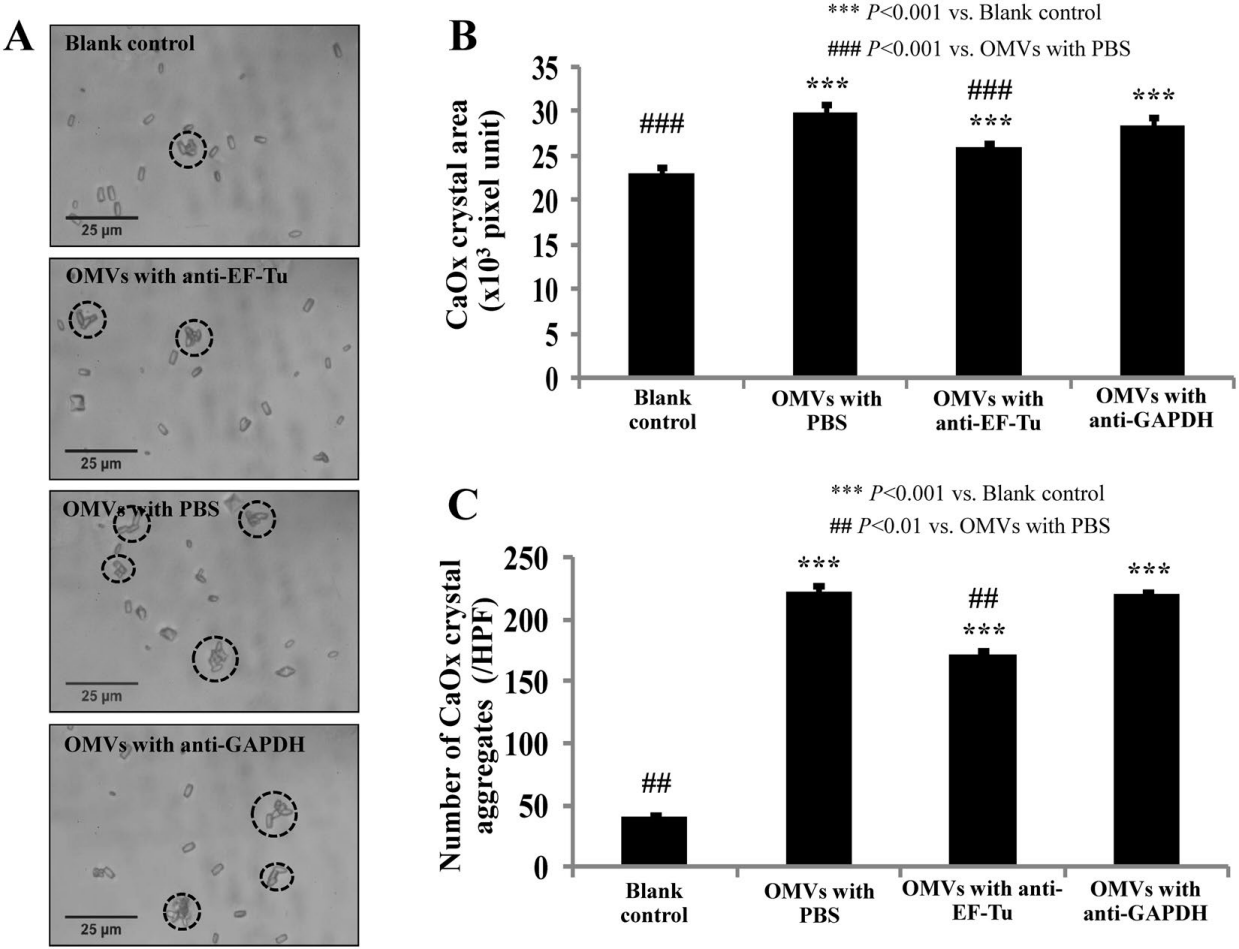
Effect of OMVs derived from EUK (without or with anti-EF-Tu antibody neutralization) on CaOx crystallization. (**A**) Crystal morphology and image analysis. The circles indicate crystal aggregates. **(B)** Quantitative analysis of crystal area (representing crystal growth). **(C)** Number of crystal aggregates. Each bar represented mean ± SEM of the data obtained from three independent experiments. HPF = high-power field.

The effects of EF-Tu on OMVs derived from EUK to CaOx crystallization, crystal growth, and crystal aggregation were first addressed by crystallization assay followed by crystal image analysis. The results showed that CaOx crystallization with EUK-derived OMVs caused significant increase in crystal size (which reflected crystal growth) and number of crystal aggregates as compared to the blank control (Fig. 5). When these OMVs were neutralized by monoclonal anti-EF-Tu antibody, such increases were significantly lower, whereas neutralization by isotype (anti-GAPDH) antibody did not affect such increases induced by EUK-derived OMVs (Fig. 5).

**Figure 6:**
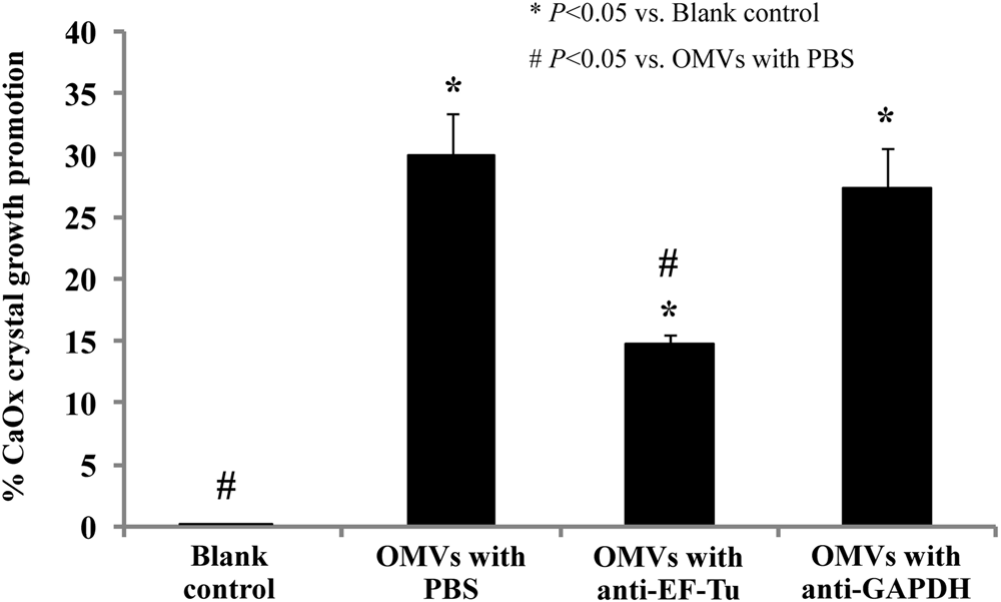
Effect of OMVs derived from EUK (without or with anti-EF-Tu antibody neutralization) on CaOx crystal growth as determined by spectrophotometric oxalate-depletion assay. % CaOx crystal growth promotion = [(C − T)]/C × 100, where C was the rate of free oxalate depletion in the blank control and T was the rate of free oxalate depletion under each of the OMVs conditions tested. Each bar represented mean ± SEM of the data obtained from three independent experiments.

The promoting effects of EF-Tu on EUK-derived OMVs to CaOx crystal growth was also confirmed by the spectrophotometric oxalate-depletion assay. The data revealed that EUK-derived OMVs had CaOx crystal growth promoting activity (approximately 30%) as compared to the blank control (Fig. 6). When these OMVs were neutralized by monoclonal anti-EF-Tu antibody, the OMVs-induced CaOx crystal growth promoting activity was significantly reduced by a half, whereas neutralization by isotype (anti-GAPDH) antibody did not affect such crystal growth promotion induced by EUK-derived OMVs (Fig. 6).

**Figure 7:**
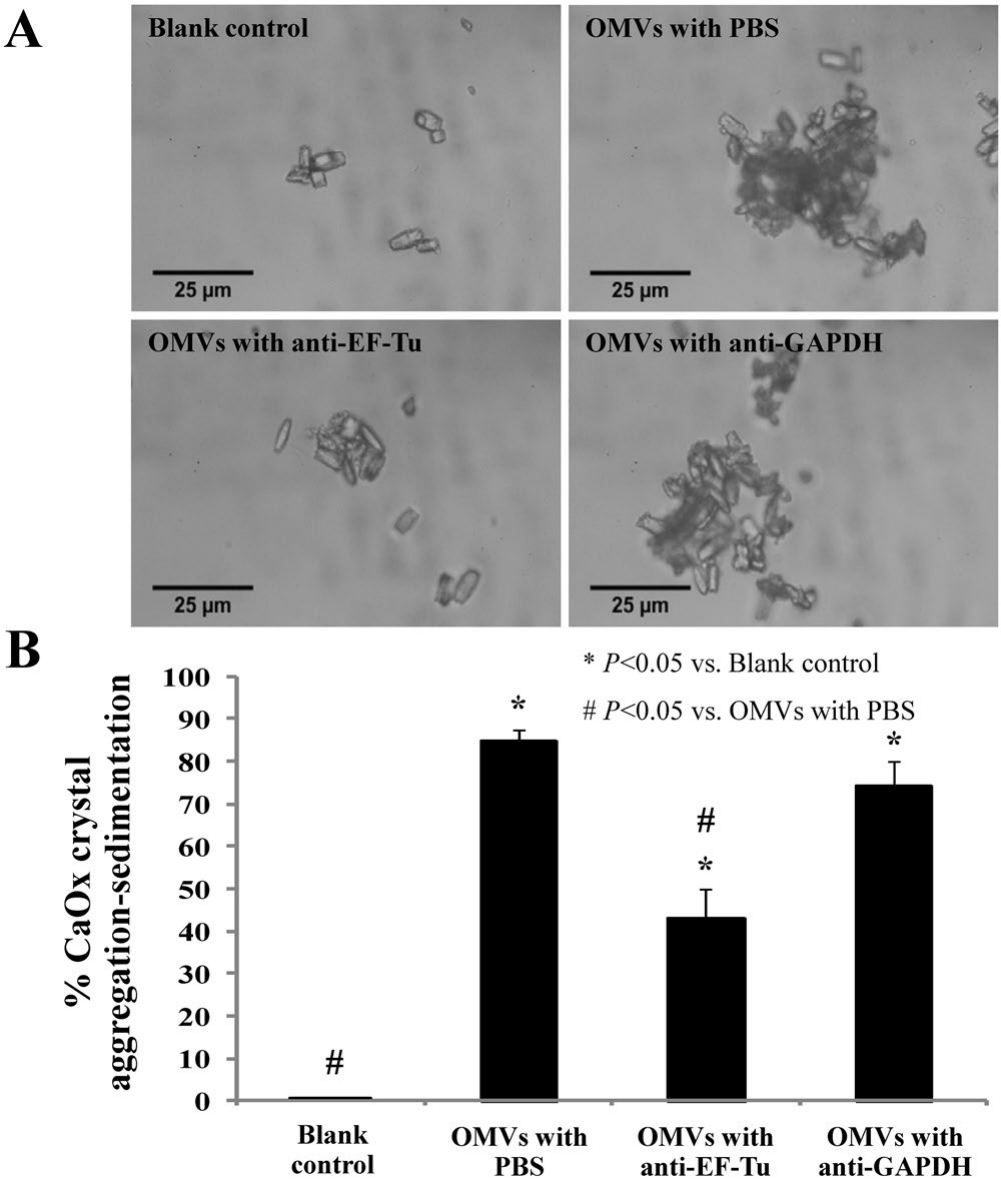
Effect of OMVs derived from EUK (without or with anti-EF-Tu antibody neutralization) on CaOx crystal aggregation as determined by crystal aggregation-sedimentation assay. (**A**) Morphology of CaOx crystal aggregates occurred in each condition. (**B**) % CaOx crystal aggregation-sedimentation = [(A − T)]/A × 100, where A was the rate of reduction in the solution turbidity of the blank control and T was the rate of reduction in the solution turbidity under each of the OMVs conditions tested. Each bar represented mean ± SEM of the data obtained from three independent experiments.

The CaOx crystal aggregation-sedimentation study was performed to confirm the promoting effects of EF-Tu on EUK-derived OMVs to CaOx crystal aggregation. The data revealed that EUK-derived OMVs caused approximately 85% increase of CaOx crystal aggregation-sedimentation as compared to the blank control (Fig. 7). When these OMVs were neutralized by monoclonal anti-EF-Tu antibody, the OMVs-induced increase of crystal aggregation-sedimentation was significantly reduced by a half, whereas neutralization by isotype (anti-GAPDH) antibody did not affect such increase induced by OMVs (Fig. 7).

**Figure 8:**
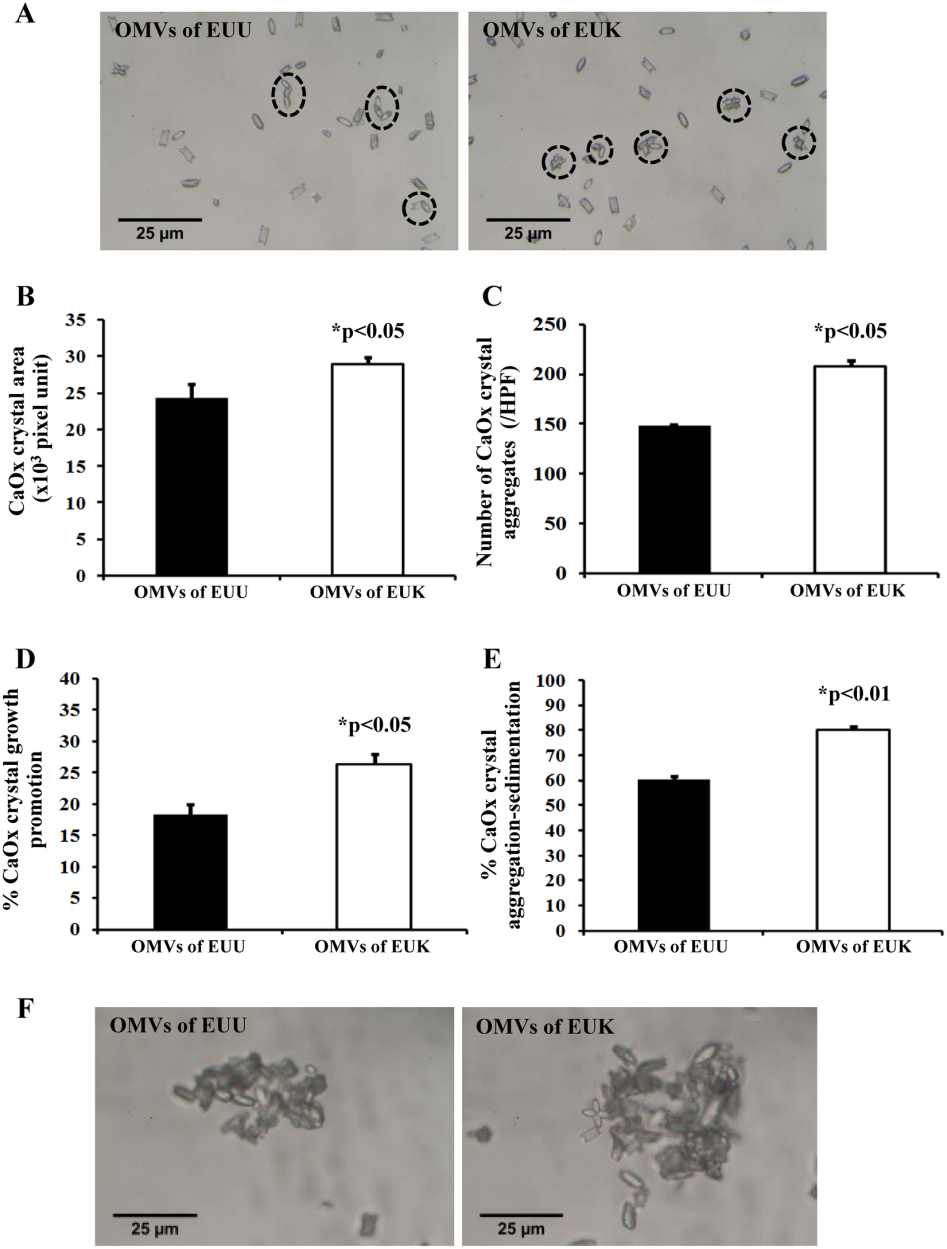
Differential effects of OMVs derived from EUU vs. EUK on CaOx crystallization, growth and aggregation. **(A)** Crystal morphology and image analysis. The circles indicate crystal aggregates. **(B)** Quantitative analysis of crystal area (representing crystal growth). **(C)** Number of crystal aggregates. **(D)** CaOx crystal growth as determined by spectrophotometric oxalate-depletion assay. **(E)** CaOx crystal aggregation as determined by crystal aggregation-sedimentation assay. **(F)** Morphology of CaOx crystal aggregates occurred in each condition. Each bar represented mean ± SEM of the data obtained from three independent experiments.

**Figure 9:**
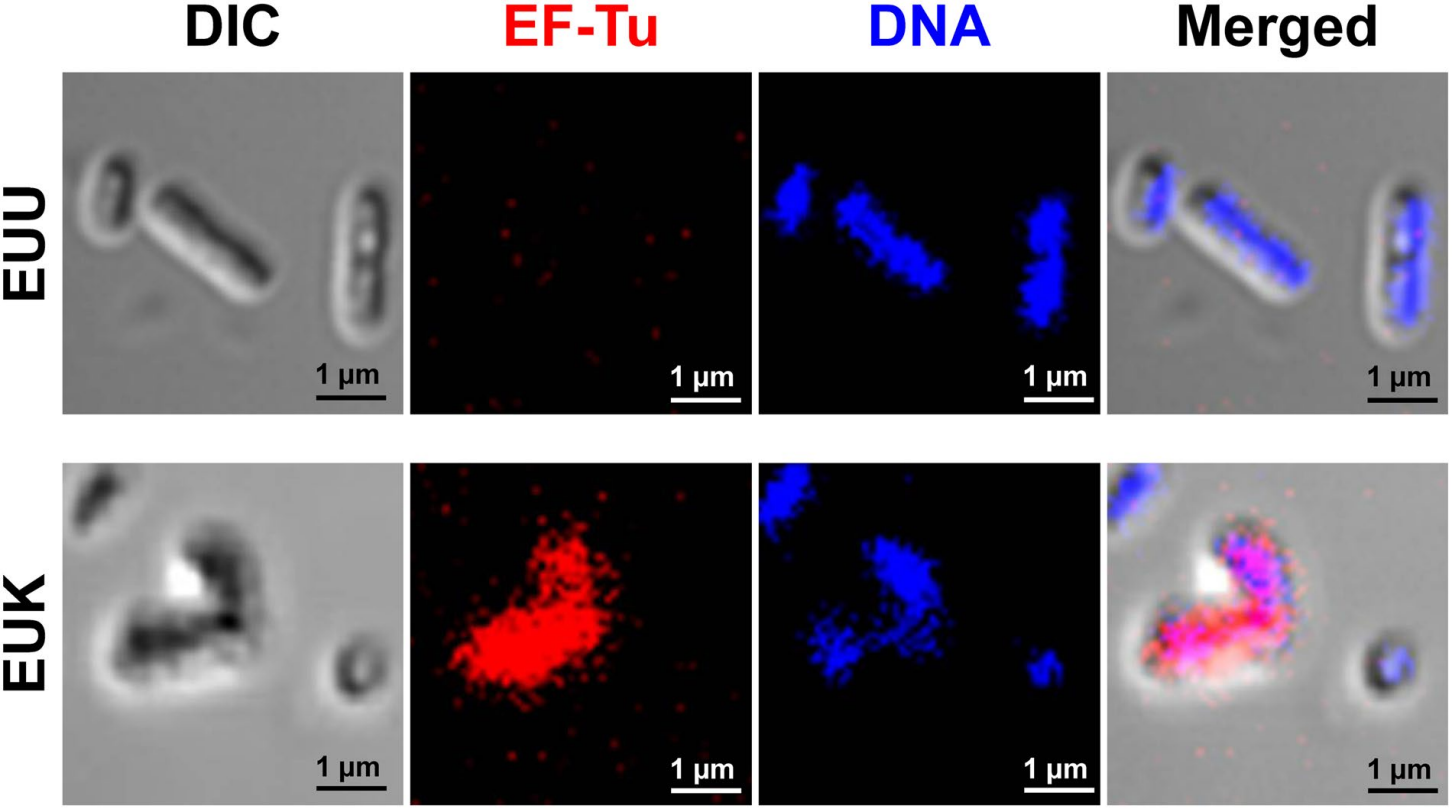
Immunofluorescence staining of EF-Tu on bacterial cell surface of EUU vs. EUK. Bacterial cells were fixed with 4% paraformaldehyde (without permeabilization as to stain only the surface EF-Tu, not its cytoplasmic form) and then incubated with mouse monoclonal anti-EF-Tu antibody followed by incubation with Alexa Fluor 555-conjugated donkey anti-mouse secondary antibody containing 0.1 μg/ml Hoechst dye for DNA staining. The immunofluorescence micrographs were then obtained using ECLIPSE Ti-Clsi4 Laser Unit (Nikon) equipped with NIS-Elements D V.4.11 (Nikon). Original magnification power was 630X for all panels. Expression of surface EF-Tu is shown in red, whereas DNA contents are illustrated in blue. DIC = differential interference contrast mode of image capture.

Additionally, differential effects of OMVs derived from EUU vs. EUK on CaOx crystallization, growth and aggregation were also evaluated. The data showed that crystal size, growth, and aggregation were significant greater when CaOx crystals were incubated with EUK-derived OMVs as compared to those derived from EUU (Fig. 8). Moreover, immunofluorescence staining of EF-Tu on bacterial cell surface was performed without permeabilization (to allow the antibody to conjugate only with EF-Tu on the cell surface, whereas only Hoechst dye could penetrate through the cells to bind with DNA for cellular localization). The immunofluorescence staining of surface EF-Tu confirmed the greater expression level of surface EF-Tu on EUK as compared to EUU (Fig. 9).

All these results suggested that OMVs derived from EUK could promote CaOx crystallization, crystal growth and crystal aggregation, and EF-Tu on OMVs was responsible, at least in part, for such promoting effects. These data were in accordance with those reported in a previous study demonstrating that EF-Tu of *B. subtilis* can bind to calcium ions^22^. In addition, Day and coworker^23^ have shown that EF-Tu also has EF-hand domains via the genomic analysis of the EF-hand related sequences in *Arabidopsis*. Thus, the promoting effects of EF-Tu on CaOx crystal aggregation may be mediated by EF-hand calcium-binding domains, which may be able to bind with several COM crystals to form CaOx crystal aggregate/agglomerate.

Our findings were in concordance with those reported in previous *in vitro* and *in vivo* studies indicating that *E. coli* plays significant roles in CaOx stone pathogenesis^5–7^. However, it should be noted that we examined EF-Tu and OMVs derived from only *E. coli* in the present study. Whether they play significant roles in the pathogenesis of struvite stones induced by *Proteus* species and other urease-producing bacteria remain unclear and should be further elucidated. Additionally, although neutralization with monoclonal anti-EF-Tu antibody could reduce the promoting activity of OMVs on CaOx crystallization, crystal growth and crystal aggregation, such reduction was only partial. Therefore, it is thus plausible that there are other active molecules on OMVs’ surface, in addition to EF-Tu, that can also exhibit such promoting activities. Characterizations of all these active molecules on OMVs and in other subcellular fractions as well as secreted products of *E. coli* should be further done and will lead to a more complete picture of the mechanisms underlying promoting activities of *E. coli* in kidney stone pathogenesis.

## Conclusions

Our present study has demonstrated that EUU and EUK with identical phenotypic characteristics and antimicrobial susceptibility patterns had differential proteome expression. Among the common differentially expressed proteins identified, the greater level of EF-Tu in EUK was validated by Western blotting. OMVs derived from EUK had greater promoting activities on CaOx crystallization, crystal growth and aggregation as compared to those derived from EUU. Neutralizing the OMVs of EUK with monoclonal anti-EF-Tu antibody, not with an isotype antibody, significantly reduced all these OMVs-induced promoting effects. Moreover, immunofluorescence staining of EF-Tu on bacterial cell surface confirmed the greater expression of surface EF-Tu on EUK. Our data indicate that surface EF-Tu and OMVs play significant roles in promoting activities of *E. coli* on CaOx crystallization, crystal growth and aggregation.

## Materials and Methods

### Ethics

All human subjects and clinical specimens were conducted in accordance with the Declaration of Helsinki and written informed consent was obtained from each of the participants. This study was approved by the Institutional Ethical Committee of Khon Kaen University, Khon Kaen, Thailand (approval no. HE 521177, HE 551307 and HE 581501).

### Sample collection and selection criteria

Bacterial culture was performed for urine samples collected from a total of 100 kidney stone formers admitted to Khon Kaen Hospital for elective kidney stone removal by percutaneous nephrolithotomy during 2009–2010. Note that inclusion and exclusion criteria as well as stone analysis were detailed in our previous report^5^. Among them, 36 had positive bacterial cultures from their urine and/or stone samples and 9 had *E. coli* isolated from their urine (namely EUK). EUU were isolated from urine of 200 UTI patients admitted at Srinagarind Hospital, Khon Kaen University during 2012–2013. The inclusion/exclusion criteria in the latter group were as follows: (i) All subjects were born and resided in the northeastern region of Thailand (same as the EUK group); (ii) All subjects had signs and symptoms of upper tract UTI with no history of kidney stone disease, renal failure, and kidney tumors from clinical investigations including X-ray, plain KUB, abdominal ultrasound, and intravenous pyelography (IVP); and (iii) Positive urine culture for pure *E. coli* with bacterial colony count >10^5^ colony forming unit (CFU)/mL.

### Antimicrobial susceptibility test

All *E. coli* isolates were tested for antimicrobial susceptibility using the disc diffusion method as described in details in our previous study^5^. The antimicrobial agents that were tested included amikacin (AK, 30 μg), ampicillin (AMP, 10 μg), cephalothin (CF, 30 μg), cefotaxime (CTX, 30 μg), ceftazidime (CAZ, 30 μg), sulfamethoxazole/trimethoprim (SXT, 1.25/23.75 μg), gentamicin (GM, 10 μg), norfloxacin (NOR, 10 μg) and ofloxacin (OFX, 5 μg) (Oxoid Ltd.; Basingstoke, UK). Extended spectrum β-lactamase (ESBL) production was also determined by the double-disc diffusion test according to the standard method of the Clinical and Laboratory Standards Institute.

### Determination of bacterial colony size

Colony size of *E. coli* isolates were determined following the method described by Smanthong *et al*.^24^. Briefly, a single colony of each *E. coli* isolate was cultivated in tryptic soy broth (TSB) (Oxoid Ltd.) for 24 h. The suspension of *E. coli* was adjusted to 0.5 McFarland standards. The suspension (50 μL) from each sample was spread onto MacConkey agar (Oxoid Ltd.) and incubated at 37 °C for 24 h. The diameters of all the colonies on the spread plate were measured with a Vernier caliper.

### Determination of bacterial cell length

A single colony from the spread plate that had been used to measure the colony size was picked up with a sterile needle and suspended in 1 μL distilled water. The bacterial suspension of *E. coli* was smeared on a glass slide and stained with 0.25% safranin O. The lengths of individual *E. coli* cells were measured under a light microscope (Nikon ECLIPSE 80i Microscope, Nikon Corporation; Tokyo, Japan).

### Time to the mid-log phase of the bacterial growth curve

A single colony of each *E. coli* isolate from the MacConkey agar was suspended and adjusted to 0.5 McFarland standards in 1 mL of TSB. The bacterial suspension was inoculated in each well of a 96-well plate (200 μL/well) (NunclonTM Delta Surface, Thermo Fisher Scientific; Jiangsu, China). The optical density at λ570 nm was recorded every 15 min for 12 h by a Tecan Sunrise plate reader (Tecan; Vienna, Austria).

### Preparation of whole cell lysate

All *E. coli* isolates were cultivated in 50 mL TSB at 37 °C overnight. The bacterial cells were then harvested by centrifugation at 4,500 × g for 10 min and washed twice with low-salt phosphate buffered saline (PBS) (3 mM KCl, 1.5 mM KH_2_PO_4_, 68 mM NaCl, and 9 mM NaH_2_PO_4_). The pellets were resuspended in a 2-D lysis buffer containing 7 M urea, 2 M thiourea, 4% (w/v) 3-[(3- cholamidopropyl)dimethyl-ammonio]-1-propanesulfonate (CHAPS), 2% (v/v) ampholytes (pH 3–10) and 40 mM dithiothreitol (DTT), and incubated at 4 °C for 30 min. Unsolubilized debris was removed by centrifugation at 20,000 × g and 4 °C for 10 min. Protein concentrations were determined by the Bradford method.

### Two-dimensional gel electrophoresis (2-DE)

Proteins derived from each *E. coli* isolate were resolved by 2-DE according to protocols published previously^25, 26^ with some modifications (n = 3 gels derived from each *E. coli* isolate; a total of 24 gels were analyzed). Briefly, equally loaded proteins (200 μg/sample) were premixed with a rehydration buffer containing 7 M urea, 2 M thiourea, 2% (w/v) CHAPS, 0.5% (v/v) ampholytes (pH 3–10), 18 mM DTT and 0.002% bromophenol blue (to make the final volume of 250 μL), and then rehydrated onto Immobilized pH gradient (IPG) strip (linear pH 3–10, 13-cm-long) (GE Healthcare; Uppsala, Sweden). The first-dimensional separation was performed in an Ettan IPGphor II Isoelectric Focusing Unit (GE Healthcare) at 20 °C, using the stepwise mode to reach 17,000 Vh. After completion of the isoelectric focusing (IEF), the strip was first equilibrated for 15 min in an equilibration buffer containing 6 M urea, 65 mM DTT, 29.3% glycerol, 75 mM Tris-HCl (pH 8.8), 2% sodium dodecyl sulfate (SDS) and 0.002% bromophenol blue, and then in another similar buffer that replaced the DTT with 135 mM iodoacetamide, for another 15 min. The second dimensional separation was performed by 12% SDS-PAGE using a SE 600 Ruby (GE Healthcare) with a constant electric current of 25 mA/gel for approximately 4 h. The resolved protein spots were stained by colloidal Coomassie brilliant blue G-250 (AppliChem; Darmstadt, Germany) at room temperature (RT) for 18 h and then visualized using ImageScanner III (GE Healthcare).

### Matching and quantitative intensity analysis

The Image Master 2D platinum software (GE Healthcare) was used for the matching and analysis of the protein spots across individual 2-D gels. Parameters used for spot detection were: (i) minimal area = 20 pixels, (ii) smooth factor = 3 and (iii) saliency = 150. The reference gel for each isolate was created from an artificial gel by combining all of the spots present on all the triplicate gels obtained from each sample into one image. This reference gel was used for determination of the existence and differences in levels of proteins expressed in each pair of EUU vs. EUK that had identical antimicrobial susceptibility pattern. Background subtraction was performed and the intensity volume of each spot was normalized with the total intensity volume (summation of the intensity volumes obtained from all spots within the same 2-D gel). The intensity volume of each corresponding protein spot matched across different gels in EUU vs. EUK was then compared. Significant differences consistent in all pairs of EUU vs. EUK with identical antimicrobial susceptibility pattern were considered “common changes” among all pairs and were then subjected to in-gel tryptic digestion and identification by tandem mass spectrometry (MS/MS).

### In-gel tryptic digestion and protein identification by nanoLC-MS/MS

The common differentially expressed proteins were excised from 2-D gels and subjected to in-gel tryptic digestion as described previously^27^. Separation of the digested peptides was performed using an EASY-nLC II (Bruker Daltonics; Bremen, Germany). Briefly, peptides were loaded from a cooled (7 °C) auto sampler into an in-house, 3-cm-long pre-column containing 5-μm C18 resin (Dr. Maisch GmbH; Ammerbuch, Germany) and then into a 10-cm-long analytical column packed with 3-μm C18 resin (Dr. Maisch GmbH) using mobile phase A (0.1% formic acid). The peptides were then separated with a mobile phase B (acetronitrile/0.1% formic acid) gradient elution with three steps as follows: 0–35% for 30 min, 35–80% for 10 min and then 80% for 10 min at a flow rate of 300 mL/min. The peptide sequences were then analyzed with an amaZonspeed ETD (Bruker Daltonics) with ESI nanosprayer ion source (spray capillary: fused silica with outer diameter of 90 μm and inner diameter of 20 μm) controlled by HyStar version 3.2 and trapControl version 7.1. The mass spectrometric parameters were set as follows: electrospray voltage = 4,500 V, high-voltage end-plate offset = 500 V, nebulizer gas = 0.55 bar, dry gas = 5.0 l/min and dry temperature = 150 °C. The precursors were scanned from the 400 to 2,200 m/z range with the enhanced resolution mode (speed = 8,100 m/z/s), ICC (Ion Charge Control) target = 200000 and maximal accumulation time = 50 ms. The three most intense signals in every MS scan were selected for MS/MS analysis, whereas singly charged ions were excluded. For the MS/MS experiments, fragmented peptides from the 150 to 3,000 m/z range were scanned with the XtremeScan mode (speed = 52,000 m/z/sec), ICC target = 200,000 and maximal accumulation time = 100 ms. Mass spectra were de-convoluted via Data Analysis version 4.0 SP5 (Bruker Daltonics) to a.mgf file. Mascot software version 2.4.0 (Matrix Science; London, UK) was used to search the MS/MS spectra against the NCBI database of bacteria with the following standard Mascot parameters for CID: enzyme = trypsin, maximal number of missed cleavages = 1, peptide tolerance =  ±1.2 Da, MS/MS tolerance =  ±0.6 Da, fixed modification = carbamidomethyl (C), variable modification = oxidation (M), charge states = 2+ and 3+ and instrument type = ESI-Trap.

### Validation of the proteomic data by Western blot analysis

Proteins with an equal amount (10 μg from each sample) were resolved by 12% SDS-PAGE at 150 V for approximately 2 h using a SE260 Mini-vertical Electrophoresis Unit (GE Healthcare). The resolved proteins were then transferred onto a PVDF membrane using the iBot dry blotting system (Invitrogen; Carlsbad, CA). Nonspecific bindings were blocked with 5% (w/v) skim milk in PBS at RT for 1 h. The membrane was incubated with mouse monoclonal anti-EF-Tu (Hycult biotech; Uden, The Netherlands) (1:5,000 in 1% (w/v) skim milk/PBS) at 4 °C overnight. After washing, the membrane was further incubated with rabbit anti-mouse IgG conjugated with horseradish peroxidase (Southernbiotech; Birmingham, AL) (1:10,00αn 1% (w/v) skim milk/PBS) at RT for 1 h. For loading control, the membrane was incubated with goat polyclonal anti-GAPDH antibody conjugated with horseradish peroxidase (Abcam; Cambridge, UK) (1:300 in 1% (w/v) skim milk/PBS) at 4 °C overnight. Immunoreactive protein bands were developed with Amersham ECL Prime Western blot detection reagent (GE Healthcare) and visualized with ImageQuant Las 4000 (GE Healthcare). Band intensity was measured by ImageQuant TL software (GE Healthcare).

### Outer membrane vesicles (OMVs) preparation

OMVs were isolated from EUK and EUU following the method described by Park *et al*.^18^, with a slight modification. *E. coli* was incubated in TSB in an orbital shaking incubator at 37 °C for 18 h. The bacterial cells were then removed by centrifugation at 10,000 × g for 15 min, whereas the supernatant was filtered through 0.45-μm membrane using vacuum. The filtrate was centrifuged at 150,000 × g and 4 °C for 3 h and the remaining OMVs were resuspended in PBS. To confirm the purity of isolation, EUK-derived OMVs in PBS were placed on 400-mesh copper grids coated with formvar (Electron Microscopy Sciences; Hatfield, PA) and stained with 2% uranyl acetate. Images were obtained using a transmission electron microscope (TEM) (JEOL JEM1010, Nikon electronic Inc.; Tokyo, Japan) at an accelerating voltage of 100 kV. The expression of EF-Tu on OMVs was also confirmed by Western blot analysis as described in details above.

### Effect of EUK-derived OMVs (without or with anti-EF-Tu antibody neutralization) on CaOx crystallization

CaOx crystallization assay was performed following the method described previously^28, 29^. Briefly, CaCl_2_·2H_2_O was mixed with Na_2_C_2_O_4_ in a buffer containing 1 mM Tris-HCl buffer (pH 7.4) and 90 mM NaCl to make final concentrations of 5.0 and 0.5 mM, respectively, in a 24-well polystyrene disposable cell culture cluster with a lid (Nunclon™ Delta Surface, Thermo Fisher Scientific; Jiangsu, China). Then, an equal volume (50 μL) of PBS (blank control), 0.2 μg EUK-derived OMVs in PBS, 0.2 μg EUK-derived OMVs with 1.25 μg anti-EF-Tu in PBS, or 0.2 μg EUK-derived OMVs with 1.25 μg anti-GAPDH in PBS was added into 200 μL CaOx suspension. After 1-h incubation at RT, crystals in each condition were examined and imaged under an inverted light microscope (CKX41, Olympus; Tokyo, Japan) connected to a digital camera. The crystal size was then measured from 100 randomized individual crystals and then averaged for each OMVs condition using ImageJ software (version 1.48b) (http://imagej.nih.gov/ij/). The number of crystal aggregates, which were defined as the assembly of two or more individual crystals tightly joined together^6^, was then counted. These experiments were done in triplicate.

### Effect of EUK-derived OMVs (without or with anti-EF-Tu antibody neutralization) on CaOx crystal growth as determined by spectrophotometric oxalate-depletion assay

Effect of EUK-derived OMVs on CaOx crystal growth was evaluated by spectrophotometric oxalate-depletion assay at RT following the method described previously^6, 30^, with a slight modification. Briefly, 1 mL equilibrated solution containing 1 mM CaCl_2_·2H_2_O, 1 mM Na_2_C_2_O_4_, 1 mM Tris-HCl buffer (pH 7.4) and 90 mM NaCl was added into a cuvette. Then, an equal volume (50 μL) of PBS (blank control), 0.2 μg EUK-derived OMVs in PBS, 0.2 μg EUK-derived OMVs with 1.25 μg anti-EF-Tu in PBS, or 0.2 μg EUK-derived OMVs with 1.25 μg anti-GAPDH in PBS was added followed by 160 μg CaOx crystals. The crystal suspension was gently mixed and its absorbance was monitored at λ214 nm (to measure free oxalate level) for 60 min using a UV-visible spectrophotometer (UV-160A, Shimadzu; Kyoto, Japan). The rate of free oxalate depletion was calculated using the baseline value and the value after 60-min incubation with each of the OMVs conditions. The relative crystal growth promoting activity was calculated using the following equation:

1 $$ \% \,CaOx\,crystal\,growth\,promotion=[(C-T)]/C\times 100$$

Where C was the rate of free oxalate depletion in the blank control and T was the rate of free oxalate depletion under each of the OMVs conditions tested. These experiments were done in triplicate.

### Effect of EUK-derived OMVs (without or with anti-EF-Tu antibody neutralization) on CaOx crystal aggregation as determined by crystal aggregation-sedimentation assay

Effect of EUK-derived OMVs on CaOx crystal aggregation was evaluated by CaOx crystal aggregation-sedimentation assay at RT according to the method described previously^6, 29^, with a slight modification. Briefly, 1 mL aggregated buffer containing 1 mM CaCl_2_·2H_2_O, 0.1 mM Na_2_C_2_O_4_, 1 mM Tris-HCl buffer (pH 7.4) and 90 mM NaCl was added into a cuvette. Then, an equal volume (50 μL) of PBS (blank control), 0.2 μg EUK-derived OMVs in PBS, 0.2 μg EUK-derived OMVs with 1.25 μg anti-EF-Tu in PBS, or 0.2 μg EUK-derived OMVs with 1.25 μg anti-GAPDH in PBS was added followed by 100 μg CaOx crystals. The CaOx crystal aggregation-sedimentation was monitored at λ620 nm for 60 min by a UV-visible spectrophotometer (UV-160A; Shimadzu). The rate of reduction in the solution turbidity was calculated using the baseline value and the value after 60-min incubation with each of the OMVs conditions. The crystal aggregation was calculated using the following equation:

2 $$ \% \,CaOx\,crystal\,aggregation=[(A-T)]/A\times 100$$

where A was the rate of reduction in the solution turbidity of the blank control and T was the rate of reduction in the solution turbidity under each of the OMVs conditions tested. These experiments were done in triplicate.

In addition, the morphology of the aggregates generated by this reaction was observed under an inverted light microscope to confirm the crystal aggregation potency of each OMVs condition.

### Differential effects of OMVs derived from EUU vs. EUK on CaOx crystallization, growth and aggregation

OMVs were prepared from both EUU and EUK culture supernatants as described above. These OMVs were then subjected to comparative analyses of their effects on CaOx crystallization, crystal growth, and crystal aggregation as described above. All these comparative analyses were done in triplicate.

### Immunofluorescence staining of EF-Tu on bacterial cell surface of EUU vs. EUK

*E. coli* from EUU and EUK isolates was smeared on a coverslip and fixed with 4% paraformaldehyde in PBS for 30 min at RT without permeabilization (as to stain only the surface EF-Tu, not its cytoplasmic form). After washing with PBS, the cells were incubated with mouse monoclonal anti-EF-Tu antibody (Hycult Biotech) at a dilution of 1:50 in 1% bovine serum albumin (BSA) with PBS at 37 °C for 30 min. The cells were extensively washed with PBS and then incubated with Alexa Fluor 555-conjugated donkey anti-mouse secondary antibody (at a dilution of 1:1,000 in 1% BSA/PBS) together with 0.1 μg/ml Hoechst dye (Sigma; St. Louis, MO) (as to stain DNA contents to localize the bacterial cells) at 37 °C for 30 min. After removal of the unbound secondary antibody and dye by washing with PBS, the coverslip was mounted onto a glass slide using 50% glycerol in PBS. The surface expression of EF-Tu was then examined by ECLIPSE Ti-Clsi4 Laser Unit (Nikon) equipped with NIS-Elements D V.4.11 (Nikon).

### Statistical analysis

Quantitative data are reported as mean ± SEM. Statistical analyses were performed using the SPSS software (version17.0) (SPSS Corporation; Chicago, IL). Differences between two groups were determined by t-test, whereas comparisons among more than two groups were performed by ANOVA. P-values of less than 0.05 were considered statistically significant.

## Electronic supplementary material

Supplementary Tables S1 and S2
