# Supplementary material for: Elongation factor Tu on Escherichia coli isolated from urine of kidney stone patients promotes calcium oxalate crystal growth and aggregation
Source: Sci Rep. 2017 Jun 7;7:2953. doi: 10.1038/s41598-017-03213-x (PMC5462744; doi:10.1038/s41598-017-03213-x)
Supplement: Supplementary file 1 — Supplementary Tables S1 and S2 [file 41598_2017_3213_MOESM1_ESM.pdf]

Elongation factor Tu on *Escherichia coli* isolated from urine of kidney stone patients promotes calcium oxalate crystal growth and aggregation

Piyawan Amimanan, Ratree Tavichakorntrakool, Kedsarin Fong-ngern, Pipat Sribenjalux, Aroonlug Lulitanond, Vitoon Prasongwatana, Chaisiri Wongkham, Patcharee Boonsiri, Jariya Umka Welbat, Visith Thongboonkerd**

*Correspondence to: ratree.t@gmail.com **(OR)** thongboonkerd@dr.com

Supplementary Table S1: Antimicrobial susceptibility patterns of all 9 EUK isolates.

Pattern	Antimicrobial susceptibility test									
	Ak	AMP	CF	CTX	CAZ	SXT	GM	NOR	OFX	ESBL
#1	S	R	R	S	S	S	S	S	S	−
#2	S	R	R	R	R	S	S	R	R	+
#3 †	S	R	S	S	S	R	S	S	S	−
#4	S	S	S	S	S	R	S	S	S	−
#5	S	R	R	S	S	R	S	S	R	−
#6	S	R	R	R	R	R	R	R	R	+
#7	S	S	S	S	S	S	S	S	S	−
#8	S	S	S	S	S	S	R	R	R	−

† There were two isolates that had the same pattern.

Abbreviations used: S = susceptibility, R = resistance, AK = amikacin, AMP = ampicillin, CF = cephalothin, CTX = cefotaxime, CAZ = ceftazidime, SXT = sulfamethoxazole/trimethoprim, GM = gentamicin, NOR = norfloxacin, OFX = ofloxacin

Supplementary Table S2: Antimicrobial susceptibility patterns of EUU isolates that identically matched with EUK group.

**Pattern**	**Antimicrobial susceptibility test**									
	**Ak**	**AMP**	**CF**	**CTX**	**CAZ**	**SXT**	**GM**	**NOR**	**OFX**	**ESBL**
**#1**	S	R	R	S	S	S	S	S	S	−
**#2**	S	R	R	R	R	S	S	R	R	+
**#3**	S	R	S	S	S	R	S	S	S	−
**#4**	S	S	S	S	S	R	S	S	S	−

Abbreviations used: S = susceptibility, R = resistance, AK = amikacin, AMP = ampicillin, CF = cephalothin, CTX = cefotaxime, CAZ = ceftazidime, SXT = sulfamethoxazole/trimethoprim, GM = gentamicin, NOR = norfloxacin, OFX = ofloxacin
